# Are self-reported fertility preferences biased? Evidence from indirect elicitation methods

**DOI:** 10.1073/pnas.2407629121

**Published:** 2024-08-13

**Authors:** Christine Valente, Wen Qiang Toh, Inuwa Jalingo, Aurélia Lépine, Áureo de Paula, Grant Miller

**Affiliations:** ^a^School of Economics, University of Bristol, Bristol BS8 1TU, United Kingdom; ^b^Erasmus School of Economics, Erasmus University Rotterdam, Rotterdam 3062, Netherlands; ^c^National Population Commission, Abuja P.M.B. 281, Nigeria; ^d^Institute for Global Health, University College London, London WC1N 1EH, United Kingdom; ^e^Department of Economics, University College London, London WC1H 0AN, United Kingdom; ^f^Institute for Fiscal Studies, London WC1E 7AE, United Kingdom; ^g^Cemmap, London WC1E 7AE, United Kingdom; ^h^Department of Health Policy, Stanford Medical School, Stanford, CA 94305; ^i^National Bureau of Economic Research, Cambridge, MA 02138-5398

**Keywords:** desired fertility, list experiment, reporting bias, unintended pregnancy, Nigeria

## Abstract

Desired fertility measures are routinely collected and used by researchers and policy makers, but their self-reported nature raises the possibility of reporting bias. In this paper, we test for the presence of such bias by comparing responses to direct survey questions with indirect questions offering a varying, randomized, degree of confidentiality to respondents in a socioeconomically diverse sample of Nigerian women (N=6,256). We find that women report higher fertility preferences when asked indirectly, but only when their responses afford them complete confidentiality, not when their responses are simply blind to the enumerator. Our results suggest that there may be fewer unintended pregnancies than currently thought and that the effectiveness of family planning policy targeting may be weakened by the bias we uncover. We conclude with suggestions for future work on how to mitigate reporting bias.

About half of all pregnancies in the world are considered unintended (1), which motivates a vast global family planning effort to address persistently high unmet needs for contraception (totaling a 2021 expenditure of US$4.2 billion across all low- and lower-middle-income countries including US$576.7 from the United States alone 2). A woman who does not want to become pregnant but is not using contraception is considered as having an unmet need for family planning and to be particularly at risk of unintended pregnancy. However, the self-reported nature of fertility desires raises the possibility of bias and the size and direction of this bias, if it exists, can have major implications for our understanding of unmet need. In particular, if fertility desires are underreported, then unmet need may be lower than it seems. Biases stemming from recall error and ex post rationalization can be addressed by asking survey respondents about their desired future—rather than past fertility (3, 4), which is now the standard approach. But there is growing evidence in other domains that survey responses and even behaviors can be influenced by what others may think about these answers and behaviors(5–8). It is therefore important to test for the presence of such biases in the context of stated fertility desires.

Expressing their true fertility desires may lead survey respondents to go against what they perceive to be expected from them by family members, their community, the people asking them questions, or society at large. This may lead them to distort their answers, in a direction that is unclear a priori. Women may overstate their desired fertility if husbands want more children than their wives (9) or if they want to signal adherence to traditional values. But women may instead understate their desired fertility if they perceive this as being the answer that is preferred by the interviewer, research stakeholder, or if they wish to signal “modernity.” As put by a rural Kano resident, for instance, “everyone is now civilized and wants to rest [i.e., pause fertility]. They [i.e., researchers] think people in villages are still not civilized.”

To shed light on the sign and size of potential biases, we test, in a socioeconomically and culturally diverse sample of Nigerian women, whether respondents distort their answers to a prospective desired fertility question by comparing answers to a direct question and to two indirect questioning approaches in which we vary experimentally the degree of response confidentiality.

## Results

### Comparing Direct and Indirect Desired Fertility.

In a first survey of 6,256 cohabiting women aged 18 to 45 carried out in 2022 across five states of Nigeria, we randomize whether the respondent answers an indirect question such that her answer 1) is blind to the interviewer but not to the researcher (“colorbox” method 10) or 2) cannot be connected to her at all (using the popular “list experiment” method 8, 11). In a partial follow-up survey in 2023, we reinterviewed a random sample of 897 “colorbox” respondents and administered a list experiment. All respondents were also asked the direct prospective question about whether they wish to avoid getting pregnant to allow comparisons.

Direct responses and interviewer-blind responses are similar (2022 Sample: ${\bar{\text{Avoid}}}_{\text{Direct}}=0.640$ 95% CI: 0.622 to 0.657, N= 2,904, ${\bar{\text{Avoid}}}_{\text{ColorBox}}=0.629$ 95% CI: 0.611 to 0.647, N= 2,879; difference = 0.011, bootstrap two-sided test *P*= 0.078).

On the contrary, direct responses differ significantly from fully confidential responses. The difference between direct and list-based prevalence is indeed positive and statistically significant in each of the two alternative lists used in the 2022 survey (${\bar{\text{Avoid}}}_{\text{Direct}}=0.652$ 95% CI: 0.635 to 0.668, N= 3,352 compared to List 1: ${\bar{\text{Avoid}}}_{\text{ListExperiment}}=0.489$ 95% CI: 0.437 to 0.540, N= 3,353, difference = 0.163, bootstrap two-sided test *P*< 0.001 and compared to List 2: ${\bar{\text{Avoid}}}_{\text{ListExperiment}}=0.569$ 95% CI: 0.520 to 0.619, N= 3,353, difference = 0.082, bootstrap two-sided test *P*= 0.001). It is also positive in the 2023 survey, although we lack statistical power to reject the null of no difference between elicitation methods in the much smaller 2023 sample alone (${\bar{\text{Avoid}}}_{\text{Direct}}=0.643$ 95% CI: 0.611 to 0.675, N= 897, ${\bar{\text{Avoid}}}_{\text{ListExperiment}}=0.562$, 95% CI: 0.457 to 0.667, N= 897 (one list experiment implemented per respondent), difference = 0.081, bootstrap two-sided test *P*= 0.136).

Pooling together respondents who participated in a list experiment in either 2022 or 2023, we confirm that respondents were less likely to express the desire to avoid a pregnancy when asked fully confidentially (Pooled sample: ${\bar{\text{Avoid}}}_{\text{Direct}}=0.650$ 95% CI: 0.635 to 0.664, N= 4,249, ${\bar{\text{Avoid}}}_{\text{ListExperiment}}=0.533$ 95% CI: 0.504 to 0.562, N= 7,601, difference = 0.117, bootstrap two-sided test *P*< 0.001), suggesting that 21.9% (95% bootstrap two-sided CI: 15.4 to 28.4) more women seem to be potentially exposed to the risk of unintended pregnancy when using direct questioning than they actually are.

Pooling list 1 responses obtained in 2022 and 2023, the difference between list 1 prevalence and list 2 prevalence is 0.066 and is marginally significant (*P*-value: 0.089), calling for some caution despite reassuring additional consistency checks reported in *SI Appendix*, section S1.2. Statistically significant differences between list experiment estimates and direct responses are robust to comparison to either list.

### Implications for Policy Targeting.

**Fig. 1.**
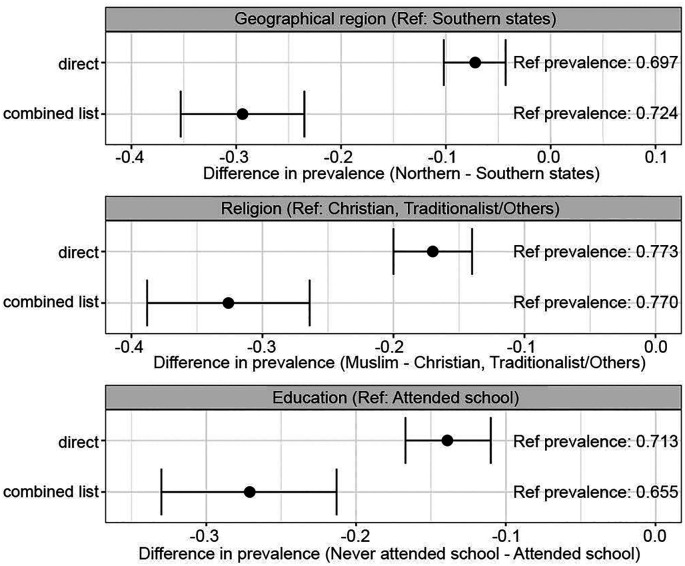
Intergroup differences are understated by direct questions. Note: The figures are generated using the pooled sample of respondents who participated in any list experiment in either 2022 or 2023. “Ref” refers to the reference category of the subgroup. “Ref prevalence” shows the proportion of reference category respondents who said they want to avoid pregnancy. Number of individuals in each subgroup: Southern States: 1510, Northern States: 2739, Christian, traditionalists/Others: 1161, Muslim: 3087, Attended School: 2311, and Never Attended School:1937.

Accurate data are needed to allocate contraceptive services and supplies to different populations based on underlying demand for family planning. If respondent bias is negatively correlated with a respondent’s genuine desire to avoid pregnancy, then responses to direct questions about the desire to avoid pregnancy will understate actual differences between individuals, and possibly even reverse the sign of differences. Reassuringly for policies targeted on the basis of directly elicited desired fertility, we find that the sign of intergroup differences is robust to direct elicitation. But direct elicitation of fertility desires greatly understates differences between groups defined, e.g., by geographical region (difference = 0.222, 95% CI: 0.164 to 0.280, bootstrap two-sided test *P*< 0.001), religion (difference = 0.156, 95% CI: 0.097 to 0.216, bootstrap two-sided test *P*< 0.001), and education level (difference = 0.133, 95% CI: 0.075 to 0.190, bootstrap two-sided test *P*< 0.001), thereby dampening the efficiency of targeting decisions (Fig. 1).

### Reporting Bias Is Not Due to Ambivalent Fertility Desires.

**Fig. 2.**
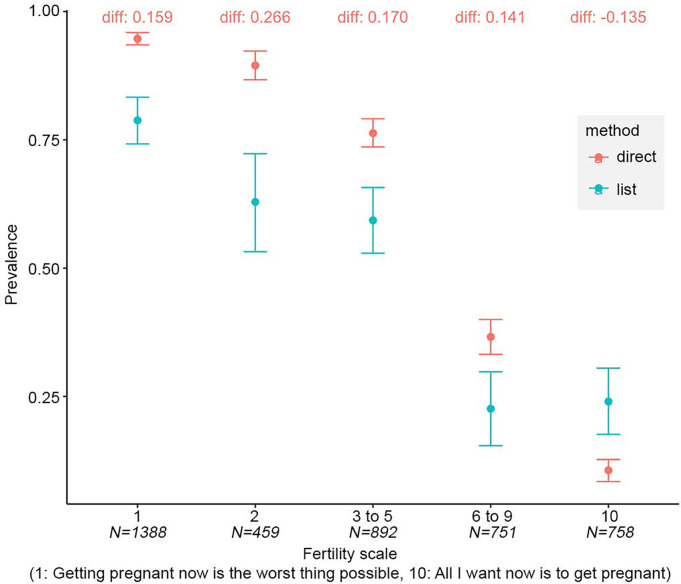
Bias is present irrespective of intensity of fertility preference. Note: The figures are generated using the pooled sample of respondents who participated in a list experiment in either 2022 or 2023. The “diff” label indicates the percentage point difference between the direct question prevalence and the combined list experiment prevalence. Using bootstrapped SEs, all differences were statistically significant (*P*< 0.05). Observations are split by quintile. “N” in the x-axis labels refer to the sample size of the quintile.

Binary questions are convenient to administer and ease classification, but many individuals are likely to have equivocal fertility desires (4). To test whether individuals who are closer to the threshold between answering “yes” or “no” are more likely to misreport, we compare direct and indirect answers to the binary fertility desire question across quintiles of women with more or less equivocal stated fertility desires based on their response to a question asking them to rate their wish to become pregnant on a scale from 1 to 10. But we do not find evidence to support the hypothesis that misreporting in binary questions is driven by women who have less clear-cut fertility desires: The absolute value of the mean difference between the direct binary question and combined list experiment in the middle quintile (0.170 pp, bootstrap two-sided test *P*< 0.001) is not much larger than differences at either extreme of the scale (difference if 1 on scale: 0.159 pp, bootstrap two-sided test *P*< 0.001; difference if 10 on scale: 0.135 pp, bootstrap two-sided test *P*< 0.001, Fig. 2).

## Discussion

Our findings indicate that responses to direct, prospective desired fertility questions may be biased, but this is not generally due to marital or social pressures to have high fertility (which would lead women to overstate fertility desires). Bias is also unlikely to be due to perceived demand from enumerators specifically since responses do not change when they are only blind to the enumerator. Instead, women appear to understate their desired fertility in response to more diffuse image concerns which are only dissipated when their answers cannot be traced back to them personally. One possible explanation consistent with both the direction of the average bias and the fact that it is more marked in low-education, rural, and Muslim subgroups, is the desire to signal the individual’s “modernity” to the researcher (but not the local enumerator).

A key policy implication of our findings is that the extent of unmet need for contraception may be lower than the prevailing wisdom suggests. Simply making answers blind to the interviewer does not appear to suffice, and a more nuanced, nonbinary direct elicitation of fertility preferences does not permit identifying women more at risk of misreporting. On the other hand, list experiments do not allow the construction of individual indicators of unmet need for contraception, as this requires linking an individual’s fertility desires with other individual variables such as sexual activity or contraceptive use. A fruitful avenue for future research would be to design a fully confidential elicitation approach which identifies the prevalence of women with “unmet need,” “met need,” or “no need” for contraception which also takes into account the possibility of bias in the direct reporting of contraceptive use. This could, in principle, be done by defining the sensitive statement of a list experiment as one stating that the respondent identifies with a woman represented in a vignette and who displays all the features of “unmet need” (or “met need” or “no need”).

Reassuringly for existing datasets and the analyses based on them, while direct questions about fertility desires may result in the inclusion of women who do not genuinely wish to avoid getting pregnant, they appear unlikely to exclude women who do.

## Materials and Methods

The direct question we investigate is “If you could fully control whether you got pregnant, and could do so without you or your partner doing anything specifically for you to avoid getting pregnant, would you personally want to **avoid** getting pregnant, at least in the next two years?”

For the direct elicitation and colorbox methods, we report the proportion of respondents answering “yes” (${\bar{\text{Avoid}}}_{\text{Direct}}$ and ${\bar{\text{Avoid}}}_{\text{ColorBox}}$, respectively) and exact binomial CIs. For subgroup effects, we use an ordinary least squares regression with robust HC3 SEs: $response_{i}=\alpha +\beta group_{i}+\epsilon_{i}$, where α gives the reference subgroup’s prevalence, while β is the difference in prevalence relative to the reference subgroup.

List experiment prevalence estimates come from a linear regression of the number of statements agreed with ($Nitems_{i,l}$) on a list l fixed effect ($\alpha_{l}$) and whether the respondent is treated for the index list ($T_{i,l}$), with SEs clustered at the respondent level:

$$\begin{array}{c} Nitems_{i,l}=\alpha_{l}+\gamma T_{i,l}+\nu_{i,l} \end{array}$$

so that ${\bar{\text{Avoid}}}_{\text{ListExperiment}}=\gamma$. To obtain subgroup effects, we run the following ordinary least squares regression:

$$\begin{array}{c} Nitems_{i,l}=\delta_{l}+\beta_{0}T_{i,l}+\beta_{1}group_{i}+\beta_{2}T_{i,l}\times group_{i}+\mu_{i,l}, \end{array}$$

where $\delta_{l}$ is a list l fixed effect, and $\beta_{0}$ is the reference subgroup’s list experiment prevalence, while $\beta_{2}$ is the difference relative to the reference subgroup. CIs of differences in prevalence across methods and *P*-values of associated tests are obtained by the bootstrap method using 1,000 iterations. Further details and robustness checks are provided in *SI Appendix*.

Institutional Review Board approval from the National Health Research Committee of Nigeria (NHREC/01/01/2007-01/03/2022) was obtained on 01/03/22 and approval from the University of Bristol School of Economics Research Ethics Committee was obtained on 06/04/22. The dataset only includes individuals who gave informed consent, thus excluding 71 subjects who declined to be interviewed.

## Supplementary Material

Appendix 01 (PDF)

## Data Availability

The anonymized datasets and code needed to replicate the analysis are available at ref. 12.
