# Supplementary material for: Are self-reported fertility preferences biased? Evidence from indirect elicitation methods
Source: Proc Natl Acad Sci U S A. 2024 Aug 13;121(34):e2407629121. doi: 10.1073/pnas.2407629121 (PMC11348086; doi:10.1073/pnas.2407629121)
Supplement: Supplementary file 1 — Appendix 01 (PDF) [file pnas.2407629121.sapp.pdf]

Are Self-Reported Fertility Preferences Biased?
Evidence from Indirect Elicitation Methods
Supporting Information

by Christine Valente, Wen Qiang Toh, Inuwa Jalingo, Aurélia Lépine,
Áureo de Paula, Grant Miller

S1: List experiment method

1 Methodology

1.1 Overview

In the list experiment, respondents are randomly assigned to a treatment and control group. The control group sees 4 non-sensitive statements, while the treatment group sees the same 4 non-sensitive statements with an additional potentially sensitive statement. The sensitive statement matches exactly our direct question about the respondent's desire to avoid pregnancy, i.e. "If you could fully control whether you got pregnant, and could do so without you or your partner doing anything specifically for you to avoid getting pregnant, personally you would want to **avoid** getting pregnant, at least in the next two years.". We effect a difference-in-means estimator using the following ordinary least squares regression to retrieve the prevalence of the fertility measure (b).

$$Nitems_i = a + bT_i + e_i$$

$Nitems_i$ is the number of statements respondent i agrees with. a is the intercept. T_i equals 1 if the respondent was allocated to the treatment group (i.e., was read a list including 4 non-sensitive statements plus the pregnancy desire statement), 0 otherwise (i.e., the respondent was read a list including 4 non-sensitive statements only). b is the treated vs. control group difference in the mean number of statements with which respondents agree, which corresponds to the rate of agreement with the "desire to avoid pregnancy" statement. Standard errors are clustered by respondent.

Estimates from a single list often have large standard errors. To improve precision, we follow best practice and employ a double list experiment in the 2022 survey. Respondents provided responses to two lists. Respondents who were assigned to the control group in the first list were assigned to the

treated group in the second list, and respondents assigned to the treated group in the first list were assigned to the control group in the second list. For double list experiment estimates, we pool the data from both lists and run the following regression with standard errors clustered at the respondent level:

$$Nitems_{i,l} = a_l + gT_{i,l} + v_{i,l}$$

, where a_l is the fixed effect of list l, $Nitems_{i,l}$ is the number of statements respondent i agrees with for list l, $T_{i,l}$ is 1 if the respondent was allocated to the treatment group for list l, 0 otherwise, and g is the prevalence of the desire to avoid a pregnancy across both lists.

1.2 Design, cognitive load, and anonymity

To ensure that respondents' answers are anonymous, the number- and choice of statements needs to be such that there are no floor or ceilings effects, i.e., very few participants would agree with none- or all of the control statements. It is therefore best practice to include one statement which all respondents should agree with and one which all respondents should disagree with, which is the case in the two lists we implemented (see results of tests for floor/ceiling effects in Section *Floor, ceiling and design effects* below).

We also sought to address potential practical issues raised by (1), which relate to the cognitive load imposed by list experiments on respondents in low-income, low-literacy/numeracy settings and which (1) effectively address through the use of, e.g., a wipe-clean handout for respondents to keep count of the statements they agree with. First, we deliberately opted for a very short questionnaire in our main 2022 survey. Second, the instructions do not ask respondents to count or recall the number of statements they agree with. Instead, the interviewer simply asks respondents to make a fist behind their back, raise a finger every time they agree with a statement, and show the interviewer their hand at the end. They also go over a simple example with the respondent to ensure that they understand. In addition, to avoid confusing respondents, we avoided unrelated statements, and focused all questions around the themes of fertility, children and family planning.

2 List experiment robustness checks

2.1 Consistency of prevalence rates across list 1 and list 2

We compared the consistency of prevalence rates across lists by using estimates and p-values from a seemingly-unrelated regression with list 1 and list 2 regressions. In 2022, when both list 1 and list 2 were implemented, the mean prevalence from the two lists used differed by 8 percentage

points (p=0.051). Pooling list 1 responses obtained in 2022 and 2023, the difference between list 1 prevalence and list 2 prevalence decreases to 0.066 but remains marginally significant (p-value: 0.089). However, we obtained consistently lower prevalence rates across the list experiments compared to direct prevalence rates of 0.652 and 0.643 in 2022 and 2023, respectively: 0.489 (list 1, 2022 survey, 95% CI: 0.437 to 0.540, N=3,353, difference with direct prevalence= 0.163, bootstrap two-sided test P<0.001), 0.569 (list 2, 2022 survey, 95% CI: 0.520 to 0.619, N=3,353, difference with direct prevalence= 0.082, bootstrap two-sided test P=0.001), and 0.562 (list 1, 2023 survey, 95% CI: 0.457 to 0.667, N=897, difference with direct prevalence=0.081, bootstrap two-sided test P=0.136).

2.2 Randomisation of lists

We tested for differences between the list experiment's treatment and control groups in the following variables: direct question about fertility preferences and sociodemographic characteristics age in years, urban/rural, education (none/primary/secondary/tertiary), ethnicity (Hausa/Yoruba/Fulani/Kanuri or Beriberi/Other), religion (Muslim/Christian/Other) and state of residence (Akwa-Ibom/Bauchi/Borno/Kano/Oyo), all measured in 2022. In 2022, differences between the list experiment's treatment and control groups were statistically insignificant, both jointly (F-statistic(16, 3318)=0.54, p=0.93) and for each individual variable. In 2023, the joint F-test remained statistically insignificant (F-statistic((15, 877)=1.23, p=0.239). although the treated group had 7.5 pp more rural women and this difference was statistically significant (p=0.024).

2.3 Floor, ceiling and design effects

Here we follow recommended tests by (2) and references cited therein. In 2022, among the control group for list 1 (list 2), 0.0% (0.0%) agreed with 0 statements and 1.2% (2.9%) agreed with 4 statements. Hence, there was no evidence of a floor effect, and very small ceiling effects. For each of list 1 and list 2, the proportion of respondents who agreed with at least X statements (where X is any number between 0 and 4) is always at least as high in the treated group than the control group. And for each of list 1 and list 2, the proportion of respondents in the treated group who agreed with at least X statements (where X is any number between 1 and 5) is never higher than those who agreed with at least X - 1 statements in the control group. Therefore, there was no detectable problematic design effect.

2.4 Randomisation between list experiments' treatment and control groups

The respondents were randomised between treated and control list groups within stratum defined by: (a) age group (18-24, 25-29, 30-34, 35-39, 40-44) and (b) polygamous, monogamous.

3 Statements used in the list experiments

List 1, Implemented in 2022 and 2023:

Treatment group

1. You hope that your children (if/when you have them) stay healthy.
2. In the past 12 months, you have read a message about family planning on a poster or heard a message about family planning on the radio.
3. You think that family planning decisions should be made jointly by husband and wife.
4. You think that 40 is a very good age for a woman to get married for the first time.
5. If you could fully control whether you got pregnant, and could do so without you or your partner doing anything specifically for you to avoid getting pregnant, personally you would want to **avoid** getting pregnant, at least in the next two years.

Control group

1. You hope that your children (if/when you have them) stay healthy.
2. In the past 12 months, you have read a message about family planning on a poster or heard a message about family planning on the radio.
3. You think that family planning decisions should be made jointly by husband and wife.
4. You think that 40 is a very good age for a woman to get married for the first time.

List 2, Implemented in 2022 only:

Treatment group

1. You think that it is good for children to learn how to read.
2. You know of a place where women can obtain a method of family planning
3. You think that a man should decide alone whether his wife can go and visit relatives
4. You hope that your children have 20 children each.
5. If you could fully control whether you got pregnant, and could do so without you or your partner doing anything specifically for you to avoid getting pregnant, personally you would want to **avoid** getting pregnant, at least in the next two years.

Control group

1. You think that it is good for children to learn how to read.
2. You know of a place where women can obtain a method of family planning
3. You think that a man should decide alone whether his wife can go and visit relatives
4. You hope that your children have 20 children each.

4 Survey instructions

Survey instructions are reported below. Participants received the same list experiment instructions in the 2022 and 2023 surveys with two exceptions. First, respondents in the 2022 survey were read the second list experiment statements after being read the first list experiment statements, whereas there was only one list experiment in the 2023 survey. Second, in the 2023 survey, due to a programming error, most *control* group respondents and a small proportion of treated group respondents received a version of the instructions which excluded the two sentences marked by "(V2:)" below.

Instructions:

I am going to read a list of statements aloud to you. Please tell me how many of these statements are true without telling me which ones are true and which ones are not (V2:, so nobody can ever know which statement applies to you and which does not).

To help you count the number of statements you agree with, please first place one hand behind your back and make a fist. If you agree with the statement I am reading, please raise one finger like this (ENUMERATOR DEMONSTRATES), keeping your hand behind your back. If you do not agree with this statement, do nothing.

Once all the statements have been read, you should have as many fingers raised behind your back as statements you agree with. At the end of reading all the statements, I will ask you to show me your hand so I know how many statements you agree with.

Here is an example. Consider the following statements:

You like papaya.
You like watermelon.
You like pineapple.

Imagine that you only like pineapple. If you heard these three statements, then you would make a fist behind your back and raise only one finger when you hear the last statement. If you liked two out of these three types of fruit, you would instead have two fingers raised after I finish reading the statements.

I will now read the statements for the study. Please (V2: do not tell me whether you agree or disagree with one particular statement or show any sign that you agree or not,) just show me your hand at the end so I know how many statements you agree with.

ASK Group X: [List 1 control group statements]

ASK Group Y: [List 1 treatment group statements]

Followed, for the 2022 survey only, by:

[LIST EXP. 2] Now I am going to read a second list of statements aloud to you. Again, please tell me how many of these statements are true without telling me which ones are true and which ones are not, so nobody can ever know which statement applies to you and which does not.

As before, please first place one hand behind your back and raise a new finger every time you agree with a sentence so once I am done reading the sentences, you will have as many fingers raised behind your back as sentences you agree with. I will now read these sentences.

ASK Group X: [List 2 treatment group statements]

ASK Group Y: [List 2 control group statements]

S2: Colorbox method

5 Methodology

The basic idea of this method is to ask respondents to answer questions using unique 6-digit codes meaning "yes" or "no", but while the respondent and the data analyst know whether the code used means "yes" or "no", the interviewer does not. This offers an appealing alternative to Audio-Computer Assisted Self-Interview (ACASI) when respondents have limited literacy and/or IT skills. More specifically, in the binary colorbox method, respondents are provided with coupons with either a black or white square attached to a 6-digit PIN code, all in a sealed envelope. The respondents are told which answer option each colored square refers to using a visual aid. To answer a question, they first chose a color coupon that corresponds to their answer to a question. They then tear off the corresponding PIN code and hand it to the enumerator. The enumerator keys it into the system. This ensures that the enumerator is blind to the answer of the respondent (each PIN code is only used once in each survey across all respondents). The researchers are able to decode individual responses as they know which PIN code corresponds to which answer option. Only researchers with access to both unanonymized respondent data and the PIN code-answer option matching would be able to trace the response back to a specific individual.

The colorbox method starts with a training phase where respondents learn how to use the coupons and receive help from the enumerator. After this training phase, the respondent does not receive any help or feedback from the enumerator | it is the "actual" colorbox interview phase. This actual phase starts with the respondent being asked to answer some easily verifiable questions (such as gender, location, etc.) on their own to check for respondent understanding during routine survey monitoring and post-survey analysis. Finally, the potentially sensitive questions of interest are asked. In the present article, the sensitive question is the question about fertility desires.

6 Colorbox robustness checks

6.1 Data entry errors

For the sensitive statement under investigation, enumerators are asked to key in the PIN code twice. This reduced the number of invalid codes to 0.3% (compared to 1.0-1.3% across the training questions, for which the enumerators had to key in the PIN code only once.)

6.2 Respondent understanding

Two verification questions were inserted in the actual colorbox section to test for respondent understanding. Only 1.1% responded incorrectly to the question "Are you more than 80 years old?" and only 2.1% responded incorrectly to "Are you less than 60 years old?".

We also made it clear to respondents that their responses would be seen by the researchers analyzing the data, but a lot of effort was exerted to ensure privacy from the enumerator and to demonstrate to respondents that enumerators did not wish to- and would not be able to see their answers. At the start of the Colorbox section, the enumerator indeed read out: "Now I am going to ask questions, but I would like you to not tell me the answers directly. Instead, I am going to give you some vouchers that will allow you to answer the questions without me knowing what the answer is. Only the people analysing the data far away from here and who have never met you will know what your answer is." In addition, respondents were told "Please be very careful when tearing the coupon along the line. It may help to fold it first. The secret code must be legible so I can read it clearly. Please never give me a code with part or all the colour code still next to it as otherwise I would know what your answer is.". They were then further prompted to "Please remember to use a secret code to answer [the enumerator's] questions rather than responding aloud.", and the enumerator reminded the respondent that she "will take away all the coupons and codes with [her] after the survey so there will be no way for [the enumerator] to work out which coupons [the respondent has] used or not used.", and the enumerator further announced that she would "turn away from [the respondent] throughout this section to provide [her] with more privacy."

7 Survey implementation

7.1 2022 survey instructions

Now I am going to ask questions, but I would like you to not tell me the answers directly. Instead, I am going to give you some vouchers that will allow you to answer the questions without me

knowing what the answer is. Only the people analysing the data far away from here and who have never met you will know what your answer is.

PHASE 1: COLORBOX TRAINING

Before we start, we will first do a training together with the training vouchers I have given you (Envelope T).

Just for this training, I will take the envelope with a T on it and I will place the coupons on the table, so that I can see if you understand the process.

I am going to ask you questions whose answer is always either YES or NO. YES corresponds to the colour white and NO to the colour black. Instead of answering me with YES or NO, I will ask you to take the coupon with the colour that corresponds to the answer, to tear it up and give me the code with the numbers.(ENUMERATOR POINTS TO THE WHITE BOX OF THE VISUAL AID AND SAYS "YES" AND POINTS TO THE BLACK BOX AND SAYS "NO". REPEATS THE OPTIONS SLOWLY TWICE).

For instance, if I ask you "Are you female?"

Find the right coupon for your answer, tear the coupon along the dotted line, and give the part with the 6-digit code to me. I will key in this code into the system like this [ENUMERATOR ENTERS 6-DIGIT CODE INTO TABLET]. And then hand it back to you like this. Please use a new coupon for the next question.

Please be very careful when tearing the coupon along the line. It may help to fold it first. The secret code must be legible so I can read it clearly and there must not be any part of the coupon colour on the part you give to me as otherwise I would know what your answer is.

Now if I ask you: "Are you male?"

Please tear the right coupon and give the 6-digit code to me. I will key in this code into the system like this [ENUMERATOR ENTERS 6-DIGIT CODE INTO TABLET]. And then hand it back to you like this. Please put the training coupons away. We will not use them further.

Now, have a try at answering two practice questions yourself, but where I can see your answers so I can tell you if you are doing it right.

1) Are we in Nigeria?

ENUMERATOR: THE RESPONDENT SHOULD TEAR A WHITE COUPON. IF NOT, SAY GENTLY: I expected you to tear a white coupon because we are in Nigeria and white means "yes".

2) Are we in Lagos?

ENUMERATOR: THE RESPONDENT SHOULD TEAR A BLACK COUPON. IF NOT, SAY GENTLY: I expected you to tear a black coupon because we are not in Lagos and black means "no".

PHASE 2: COLORBOX SECTION

Now we will begin with the real session.

Take a sealed envelope. Do not show me the contents.

You will use a new secret code for each new question. There are 8 questions in total, including two warm-up questions at the start so you can practice using the coupons without me seeing your answers. Remember that you will take away all the coupons and codes with you after the survey so there will be no way for me to work out which coupons you have used or not used.

I will turn away from you throughout this section to provide you with more privacy.

Are we ready to get started?

We will now begin with the questions.

1) Are you more than 80 years old?

2) Are you less than 60 years old?

3) If you could fully control whether you got pregnant, and could do so without you or your partner doing anything specifically for you to avoid getting pregnant, would you personally want to **avoid** getting pregnant, at least in the next two years?

... (More colorbox questions were then asked but not used in this article. Please refer to the full survey instruments uploaded as part of the submission process.)

S3: Randomisation between direct question+list experiment versus direct question+colorbox

In 2022, we randomised participants into a survey arm in which their responses were elicited via direct questioning and the list experiment, and another arm in which their responses were elicited via direct questioning and the colorbox (using simple randomisation). However we found small but statistically significant differences in some characteristics between both survey groups (overall joint F-test, $F(16, 6207) = 59.83$, $p<0.001$), such as age in years (mean diff=-0.537, p=0.003), education category (joint F-test on four education categories, $F(3, 6252) = 6.41$ $p<0.001$, largest difference: 0.027) and the probability of being Muslim (mean diff=0.028, p=0.0141). There was no statistically significant difference in responses to the direct question about the desire to avoid a pregnancy (mean diff=0.012, p=0.213). In addition, the partial follow-up was done in 2023 in part to check that the difference between list experiment prevalence and direct question prevalence was also observed

among the group answering the colorbox question in 2022 (whose answers to the colorbox question did not differ significantly from their direct answers).

S4: Further survey details

IRB approval from the National Health Research Committee of Nigeria (NHREC/01/01/2007-01/03/2022) was obtained on 01/03/22 and approval from the University of Bristol School of Economics Research Ethics Committee was obtained on 06/04/22. In this article, we focus on the following secondary objectives which formed part of a wider data collection:

1. Collect quantitative but nuanced data on the strength of women's and men's desires to avoid a pregnancy
2. Test whether women in our study sample are more likely to report a desire to delay or limit fertility when their answers to fertility intention questions are:
   (a) blind to those who are physically present during the interview (including the enumerator)
   (b) blind to everyone but themselves

8 Sampling design

To cover a diverse population, we first selected five states based on their level of unmet need for family planning among married women as of the 2018 Demographic and Health Surveys and population size. Namely, in each prevalence category from moderate (16-20%) to very high (26-30%), we selected the most populous state and, among the highest prevalence category (31-35%), we selected the two most populous states (see Figure 7.9 in 3). In each of the five selected states (Akwa Ibom, Bauchi, Borno, Kano, and Oyo), 80 enumeration areas (EAs) were randomly selected using probability weights based on their predicted population. The EAs sampling frame and predicted EA population are those used by the National Population Commission, who administered the survey on our behalf, for the 2023 Population and Housing Census of the Federal Republic of Nigeria.

In these 400 enumeration areas, a door-to-door census exercise found a total of 17,130 households. All households were visited, and after asking for consent, an adult member was asked about basic information such as gender, age and marital status of all adult household members. All cohabiting women aged between 18 and 44 who were either in a monogamous union or had at most one co-wife were invited for a short screening survey to establish their eligibility for the study.

9 Respondent recruitment and response/attrition rates

A few months after the screening exercise described above, the desired fertility survey was carried out, in parallel to a longer survey with different eligibility criteria which is beyond the scope of the present paper. A simple random sample of 65% of the women who answered the short screening survey and who, as part of this screening survey, said that they were either not pregnant or unsure of whether they were pregnant, were selected for interview with either the list experiment questionnaire (B1) or the colorbox questionnaire (B2), with equal probability. Before asking about the respondent's desired fertility, the survey instrument includes checks for whether the respondent still meets the inclusion criteria.

Starting from a sample of 9,595 surveys carried out with women identified as eligible based on the initial screening, 1,032 women could not be interviewed after three attempts, mostly because they had relocated or, more rarely, were permanently incapacitated or deceased, 210 were no longer cohabiting, and 71 did not give their consent to be interviewed, resulting in a response rate of 86%. In the successful interviews, 322 respondents no longer reported that they were aged between 18 and 45, 110 were excluded because they self-declared as infecund when asked whether they were pregnant or when asked about their fertility desires, 1,426 said they were pregnant, 150 appeared to be second interviews of women who had already been interviewed (due to technical issues in synchronizing information on the survey server), and 18 respondents did not reply to the fertility question of interest. The final analytical sample for the 2022 survey comprised 6,256 observations (2,904 colorbox respondents and 3,352 list experiment respondents).

For the 2023 survey, we selected a subsample of 2022 colorbox respondents to be interviewed in a partial follow-up. Given the other objectives of the follow-up survey and practical fieldwork considerations, the following sampling criteria were applied. First, women who had also taken part in the longer survey carried out in 2022, in addition to answering the colorbox questionnaire, were excluded from the partial follow-up sampling frame. Second, differing probabilities of re-interview were assigned to women in different states, as follows: Bauchi and Borno: 50%, Kano and Oyo: 75%, Akwa Ibom: 100%. Of the resulting sample of 1,093 women who were visited in 2023, 56 could not be interviewed after three attempts due mostly to having relocated or, more rarely, to being permanently incapacitated, deceased, or because the household was not cooperative, resulting in a response rate of 94.9%. In the successful interviews, 15 respondents were no longer cohabiting, 24 no longer reported that they were aged between 18 and 45, 3 were excluded because they self-declared as infecund when asked whether they were pregnant, 89 were pregnant at the time of the follow-up, 8 women did not reply to the fertility question of interest, and one woman had been

interviewed despite not being in the relevant sampling frame, resulting in an analytical sample of 897 (attrition rate: 17.9%, of which about half is due to pregnancy status in 2023). This moderate attrition, in addition to time elapsed and the different probabilities of re-interview by state, may have led to a change in the overall prevalence of the desire to avoid a pregnancy between the 2022 and 2023 partial follow-up samples. This prevalence is however quite stable, as documented in the main text. The direct prevalence rates were indeed 0.652 (95% CI: 0.635 to 0.668) and 0.643 (95% CI: 0.611 to 0.675) in the 2022 and 2023 samples, respectively, and the corresponding list experiments prevalence rates 0.529 (95% CI: 0.499 to 0.559, combined lists) and 0.562 (95% CI: 0.457 to 0.667).

10 SI references

1. E Kramon, K Weghorst, (Mis)measuring sensitive attitudes with the list experiment: Solutions to list experiment breakdown in Kenya. *Public Opin. Q.* 83, 236–263 (2019).

2. A Lepine, C Treibich, B d'Exelle, Nothing but the truth: Consistency and efficiency of the list experiment method for the measurement of sensitive health behaviours. *Soc. Sci. & Medicine* 266, 113326 (2020).

3. National Population Commission (NPC) [Nigeria] and ICF, *Nigeria Demographic and Health Survey 2018*. (NPC and ICF, Abuja, Nigeria, and Rockville, Maryland, USA), (2019).

11 Full questionnaires

The English version of the questionnaires used to collect the information used in this paper are included below. These were translated into Hausa and Yoruba and all three versions were deployed in the field by Computer-Assisted Personal Interviewing (CAPI) using the publicly available Census and Survey Processing System (CSPro).

FERTILITY AND HEALTH EXPECTATIONS SURVEY
UNIVERSITY OF BRISTOL

QUESTIONNAIRE B

(EACH RESPONDENT ASKED EITHER QUESTIONNAIRE B1 OR QUESTIONNAIRE B2)

**IDENTIFICATION**	
STATE:	
LOCAL GOVT. AREA:	
LOCALITY:	
ENUMERATION AREA:	
CLUSTER NUMBER:	
NAME OF HOUSEHOLD HEAD:	
HOUSEHOLD NUMBER (SAME AS IN LISTING EXERCISE):	
NAME AND LINE NUMBER OF WOMAN (FROM LISTING EXERCISE):	
TELEPHONE NUMBER 1:	TELEPHONE NUMBER 2:

INTERVIEWER'S VISITS				
	1	2	3	FINAL VISIT
DATE:				DAY ………..<br>MONTH …………<br>YEAR………..<br><br>INTERVIEW NO.<br>RESULT*
INTERVIEWER'S NAME:				
RESULT*				
NEXT VISIT: DATE<br>TIME				TOTAL NUMBER OF VISITS:

*RESULT CODES:		
1 COMPLETED	4 REFUSED	7 OTHER
2 NOT AT HOME	5 PARTLY COMPLETED	
3 POSTPONED	6 INCAPACITATED	

LANGUAGE OF QUESTIONNAIRE**:	LANGUAGE OF INTERVIEW**:	NATIVE LANGUAGE OF RESPONDENT**:	TRANSLATOR USED:
|_|_|	|_|_|	|_|_|	YES/NO
**LANGUAGE CODES: 01 ENGLISH 03 YORUBA 02 HAUSA			

	SUPERVISOR	FIELD EDITOR
NAME		
NUMBER		

INTERVIEWER: THE TEXT IN CAPITAL LETTERS IS INTENDED FOR YOU ONLY. DO NOT READ IT ALOUD.

INTRODUCTION AND CONSENT

Greetings. My name is ~~InterviewerName~~. I am working with National Population Commission. We are conducting a survey about fertility and health in five states of Nigeria. The information we collect will help researchers understand what people in these areas think about ways of planning the size of their family.

The results from this study will then be shared with the government of Nigeria and other nearby countries to inform their plans regarding family health services. You were selected for a short survey which usually takes 5-10 minutes.

ONLY FOR WOMEN SELECTED FOR THE MAIN INTERVIEW: You have also been selected for a more in-depth survey which takes about 60 minutes which I will ask you afterwards.

Information obtained from this research will be analysed by the National Population Commission and our partners from the United Kingdom and the United States. However, all of the answers you give will be confidential and no one other than members of our survey team will be able to connect your answers to you. Your answers may be shared with other researchers in the future, but in a way that does not allow them to know who responded to our survey.

You don't have to be in the survey, but we hope you will agree to answer the questions since your views are important. If I ask you any question you don't want to answer, just let me know and I will go on to the next question or you can stop the interview at any time.

Should you agree to participate, we will also like to re-interview you twice in the coming 2 years to obtain more information from you, and you may be invited to take part in a further follow-up survey in a few years' time to study changes occurring in your area.

In case you need more information about the survey, you may contact the following persons: on this card [GIVE CONSENT FORM TO RESPONDENT, ASK THE RESPONDENT TO SIGN IT, GIVE THEM THE PART WITH THE INFORMATION AND KEEP THE SIGNED PART TO GIVE TO YOUR SUPERVISOR].

National Population Commission Contact Person: Mrs Bintu Ibrahim Abba; Phone number: [redacted]. National Health Research Ethics Committee Contact Person: NHREC Chairman; Email: [redacted] Phone number: [redacted].

We also are taking measures to reduce the risk of transmission of COVID-19, including wearing face masks, keeping a distance of 2 meters from respondents to the survey, and washing our hands frequently.

INTERVIEWER: HAVE YOU READ THE INTRODUCTION AND CONSENT?

Do you have any questions?
May I begin the interview now?

<table>
<tr><td>RESPONDENT AGREES TO BE INTERVIEWED |_|</td><td>SIGNATURE OF RESPONDENT:</td><td rowspan="2"></td></tr>
<tr><td>RESPONDENT DOES NOT AGREE TO BE INTERVIEWED |_|</td><td></td></tr>
<tr><td>DATE: |__|__|__|</td><td>SIGNATURE OF INTERVIEWER:</td><td rowspan="2"></td></tr>
<tr><td></td><td></td></tr>
</table>

QUESTIONNAIRE B1

QUESTION NO./NAME	QUESTIONS AND FILTERS	CODING CATEGORIES	SKIP
SCHOOL	Have you ever attended school?<br><br>ENUMERATOR: THIS QUESTIONS REFERS TO FORMAL SCHOOLING ONLY	No……0<br>Yes…….1	→ ETHN
LEVEL	What is the highest level of school you attended:<br>primary, secondary, or higher?	Primary…..1<br>Secondary…….2<br>Higher…….3	
GRADE	What is the highest (class/year) you completed at that<br>level?<br><br>ENTER 00 IF DID NOT COMPLETE EVEN ONE YEAR AT THAT LEVEL	Class/Year |_|	
ETHN	What is your ethnic group?	1 Ekoi<br>2 Fulani<br>3 Hausa<br>4 Ibibio<br>5 Igala<br>6 Igbo<br>7 Ijaw/Izon<br>8 Kanuri/Beriberi<br>9 Tiv<br>10 Yoruba<br>96 Other<br>98 Don't know<br>999 Missing	
RELIG	What is your religion?	catholic . . . . 1<br>other christian . . . . 2<br>islam . . . . 3<br>traditionalist . . . . 4<br>other [specify: …………………] 6	
PREGCK	Are you pregnant now?	yes……1<br>no…….0<br>says she can't get pregnant…. 3<br>unsure………….8	→ IF 1 OR 3, THANK RESPONDENT AND END INTERVIEW
FERTINT_ONEQ	If you could fully control whether you got pregnant, and could do so without you or your partner doing anything specifically for you to avoid getting pregnant, would you personally want to avoid getting pregnant, at least in the next two years?	Yes…………………1<br>No…………………..0<br>SAYS SHE CAN'T GET PREGNANT 3	→ THANK RESPONDENT AND END INTERVIEW
FERTVALUE	How important would you say that it is for a woman to have children?<br><br>ENUMERATOR: READ ALL POSSIBLE ANSWERS ALLOWED AND RECORD ONLY ONE.	The most important thing a woman can do in her life…………………………1<br>Very important but not the only important thing a woman can do in her life…………2<br>Neither important nor unimportant……….3<br>Unimportant………………………………..4	
	CAPI CODING NOTES: FOR LIST EXP. 1 AND LIST EXP. 2, RESPONDENTS MUST BE RANDOMIZED INTO GROUP X AND GROUP Y WITH PROBABILITY 0.5, WITHIN 10 GROUPS DEFINED BY: AGE GROUP (18-24, 25-29, 30-34, 35-39, 40-44), POLYGAMOUS/MONOGAMOUS.		
**LIST EXP. 1**	I am going to read a list of statements aloud to you. Please tell me how many of these statements are true **without telling me which ones are true and which ones are not**, so nobody can ever now which statement applies to you and which does not.<br><br>To help you count the number of statements you agree with, please first place one hand behind your back and make a fist. If you agree with the statement I am reading, please raise one finger like this (ENUMERATOR DEMONSTRATES), keeping your hand behind your back. If you do not agree with this statement, do nothing.<br><br>Once all the statements have been read, you should have as many fingers raised behind your back as statements you agree with. At the end of reading all the		

	statements, I will ask you to show me your hand so I know how many statements you agree with.<br><br>Here is an example. Consider the following statements:<br><br>You like papaya.<br>You like watermelon.<br>You like pineapple.<br><br>Imagine that you only like pineapple. If you heard these three statements, then you would make a fist behind your back and raise only one finger when you hear the last statement. If you liked two out of these three types of fruit, you would instead have two fingers raised after I finish reading the statements.<br><br>I will now read the statements for the study. Please do not tell me whether you agree or disagree with one particular statement or show any sign that you agree or not, just show me your hand at the end so I know how many statements you agree with.		
ASK Group X:	1. You hope that your children (if/when you have them) stay healthy.<br>2. In the past 12 months, you have read a message about family planning on a poster or heard a message about family planning on the radio.<br>3. You think that family planning decisions should be made jointly by husband and wife.<br>4. You think that 40 is a very good age for a woman to get married for the first time.	NUMBER OF TRUE STATEMENTS: |__| (VALID ANSWERS: 0, 1, 2, 3, or 4)	
ASK Group Y:	1. You hope that your children (if/when you have them) stay healthy.<br>2. In the past 12 months, you have read a message about family planning on a poster or heard a message about family planning on the radio.<br>3. You think that family planning decisions should be made jointly by husband and wife.<br>4. You think that 40 is a very good age for a woman to get married for the first time.<br>5. If you could fully control whether you got pregnant, and could do so without you or your partner doing anything specifically for you to avoid getting pregnant, personally you would want to avoid getting pregnant, at least in the next two years.	NUMBER OF TRUE STATEMENTS: |__| (VALID ANSWERS: 0, 1, 2, 3, 4 or 5)	
**LIST EXP.2**	Now I am going to read a second list of statements aloud to you. Again, please tell me how many of these statements are true **without telling me which ones are true and which ones are not**, so nobody can ever now which statement applies to you and which does not.<br><br>As before, please first make a fist behind your back and raise a new finger every time you agree with a statement so once I am done reading the statements, you will have as many fingers raised behind your back as statements you agree with. I will now read these new statements.		
ASK Group X:	1. You think that it is good for children to learn how to read.<br>2. You know of a place where women can obtain a method of family planning	NUMBER OF TRUE STATEMENTS: |__| (VALID ANSWERS: 0, 1, 2, 3, 4, or 5)	

	3. You think that a man should decide alone whether his wife can go and visit relatives<br>4. You hope that your children have 20 children each.<br>5. If you could fully control whether you got pregnant, and could do so without you or your partner doing anything specifically for you to avoid getting pregnant, personally you would want to avoid getting pregnant, at least in the next two years.		
ASK Group Y:	1. You think that it is good for children to learn how to read.<br>2. You know of a place where women can obtain a method of family planning<br>3. You think that a man should decide alone whether his wife can go and visit relatives<br>4. You hope that your children have 20 children each.	NUMBER OF TRUE STATEMENTS: |__| (VALID ANSWERS: 0, 1, 2, 3, or 4)	
ANCHORING VIGNETTES<br><br>CAPI NOTES: RANDOMIZE ORDER OF VIGNETTES	This next section will require additional concentration. I will read to you some stories about people with varying levels of desire for another child.<br><br>I would like to know how you view each story and rate how much of a good or bad thing a new pregnancy would be for the person described.		
VIGNETTE 1	BOSE's youngest child is six months old and she is not sure whether she should have any more children. Money is limited and she has been struggling to buy more expensive foods like fish and meat for her family. Her husband is not sure whether he wants any more children either.<br><br>Please put yourself in BOSE's place. On a scale from 1 to 10, where 10 means that all [NAME] wants in life right now is to get pregnant and 1 means that getting pregnant now would be the worst possible thing to happen to her, what number would best reflect how she would feel about getting pregnant right now if…		
a-	… BOSE is [**RANDOM AGE 18-44] years old**?<br><br>CAPI NOTES: WHERE IT SAYS "RANDOM AGE 18-44", THE QUESTIONNAIRE SHOULD BE PROGRAMMED SO THAT A RANDOM NUMBER BETWEEN 18 AND 44 (INCLUSIVE) APPEARS IN THE QUESTION TO BE READ BY THE ENUMERATOR	RESPONSE: |_|_| (VALID RESPONSES ARE 1, 2, 3, 4, 5, 6, 7, 8, 9, OR 10)	
b-	… BOSE is [**RANDOM AGE 18-44] years old**?	SAME RESPONSE AS VIGNETTE 1a-……………..99<br>DIFFERENT RESPONSE: |_|_| (VALID RESPONSES ARE 1, 2, 3, 4, 5, 6, 7, 8, 9, OR 10)	
VIGNETTE 2	HALIMA and her husband have been trying for another child for a couple of months. They are not wealthy, but they are able to buy everything they need for their family.		

	Please put yourself in HALIMA's place. On a scale from 1 to 10, where 10 means that all [NAME] wants in life right now is to get pregnant and 1 means that getting pregnant now would be the worst possible thing to happen to her, what number would best reflect how she would feel about getting pregnant right now if…		
a-	… HALIMA is [**RANDOM AGE 18-44] years old**?	RESPONSE: |_|_| (VALID RESPONSES ARE 1, 2, 3, 4, 5, 6, 7, 8, 9, OR 10)	
b-	… HALIMA is [**RANDOM AGE 18-44] years old**?	SAME RESPONSE AS VIGNETTE 2a-……………..……………..99<br>DIFFERENT RESPONSE: |_|_| (VALID RESPONSES ARE 1, 2, 3, 4, 5, 6, 7, 8, 9, OR 10)	
VIGNETTE 3	ADA would like to have another child in the future but she would prefer to wait for a couple of years until she gets pregnant again as she finds it tiring to care for several young children at the same time.<br><br>Please put yourself in ADA's place. On a scale from 1 to 10, where 10 means that all [NAME] wants in life right now is to get pregnant and 1 means that getting pregnant now would be the worst possible thing to happen to her, what number would best reflect how she would feel about getting pregnant right now if…	RESPONSE: |_|_| (VALID RESPONSES ARE 1, 2, 3, 4, 5, 6, 7, 8, 9, OR 10)	
a-	… ADA is [**RANDOM AGE 18-44] years old**?	RESPONSE: |_|_| (VALID RESPONSES ARE 1, 2, 3, 4, 5, 6, 7, 8, 9, OR 10)	
b-	… ADA is [**RANDOM AGE 18-44] years old**?	SAME RESPONSE AS VIGNETTE 3a-……………..……………..99<br>RESPONSE: |_|_| (VALID RESPONSES ARE 1, 2, 3, 4, 5, 6, 7, 8, 9, OR 10)	
FERTSCALE	Now I would like to ask you a question about yourself using the same scale as the one you have used when commenting on the stories I have just read to you.<br><br>On a scale from 1 to 10, where 10 means that all you want in life right now is to get pregnant and 1 means that getting pregnant now would be the worst possible thing to happen to you, what number would best reflect how you would feel about getting pregnant right now?	RESPONSE: |_|_| (VALID RESPONSES ARE 1, 2, 3, 4, 5, 6, 7, 8, 9, OR 10)	
H_FERTSCALE	Now I would like to ask you a question about your husband/partner using the same scale as the one you have used when commenting on the stories I have just read to you.<br><br>On a scale from 1 to 10, where 10 means that all your husband wants in life right now is for you to get pregnant and 1 means that you getting pregnant now would be the worst possible thing to happen to your husband, what number do you think would best reflect how your husband would feel about you getting pregnant right now?	RESPONSE: |_|_| (VALID RESPONSES ARE 1, 2, 3, 4, 5, 6, 7, 8, 9, OR 10)	

Now I would like to ask a few questions about facilities in your household.

QUESTION NO./NAME	QUESTIONS AND FILTERS	CODING CATEGORIES	SKIP
WATER1	What is the main source of drinking water for members of your household?	**PIPED WATER**<br>PIPED INTO DWELLING . . . . 11<br>PIPED TO YARD/PLOT . . . . 12<br>PIPED TO NEIGHBOR . . . . 13<br>---<br>PUBLIC TAP/STANDPIPE . . . . 14<br>TUBE WELL OR BOREHOLE . . . . 21<br>**DUG WELL**<br>PROTECTED WELL . . . . 31<br>UNPROTECTED WELL . . . . 32<br>**WATER FROM SPRING**<br>PROTECTED SPRING . . . . 41 103<br>UNPROTECTED SPRING . . . . 42<br>RAINWATER . . . . 51<br>TANKER TRUCK . . . . 61<br>CART WITH SMALL TANK . . . . 71<br>SURFACE WATER (RIVER/DAM/ LAKE/POND/STREAM/CANAL/ IRRIGATION CHANNEL) . . . . 81<br>---<br>BOTTLED WATER . . . . 91<br>SACHET WATER . . . . 92<br>OTHER 96	→TOILET1<br>→TOILET1<br>→TOILET1<br>-----------------------------------<br>IF ANSWERS 14 TO 81 →WATER2<br><br>→ WATER2
WATER2	Where is that water source located?	IN OWN DWELLING . . . . 1<br>IN OWN YARD/PLOT . . . . 2<br>ELSEWHERE 3	→TOILET1<br>→TOILET1
WATER3	How long does it take to go there, get water, and come back?	|_|_|_| MINUTES<br>DON'T KNOW 998	
TOILET1	What kind of toilet facility do members of your household usually use?<br><br>IF NOT POSSIBLE TO DETERMINE, ASK PERMISSION TO OBSERVE THE FACILITY.	**FLUSH OR POUR FLUSH TOILET**<br>FLUSH TO PIPED SEWER SYSTEM . . . . 11<br>FLUSH TO SEPTIC TANK . . . . 12<br>FLUSH TO PIT LATRINE . . . . 13<br>FLUSH TO SOMEWHERE ELSE . . . . 14<br>FLUSH, DON'T KNOW WHERE . . . . 15<br>**PIT LATRINE**<br>VENTILATED IMPROVED PIT LATRINE . . . . . 21<br>PIT LATRINE WITH SLAB . . . . 22<br>PIT LATRINE WITHOUT SLAB/OPEN PIT . . . . . 23<br>COMPOSTING TOILET . . . . 31<br>BUCKET TOILET . . . . 41<br>HANGING TOILET/HANGING LATRINE . . . . 51<br>NO FACILITY/BUSH/FIELD . . . . 61<br>OTHER [SPECIFY:……………………………..]………………… …96	
TOILET2	Do you share this toilet facility with other households?	Yes……..1<br>No………0	
HHITEM	Does your household have:<br>a. Electricity?<br>b. A radio?<br>c. A television?<br>d. A non-mobile telephone?<br>e. A computer?<br>f. A refrigerator?<br>g. A table?	a. Yes……1 No……..2<br>b. Yes……1 No……..2<br>c. Yes……1 No……..2<br>d. Yes……1 No……..2<br>e. Yes……1 No……..2<br>f. Yes……1 No……..2<br>g. Yes……1 No……..2<br>h. Yes……1 No……..2<br>i. Yes……1 No……..2	

	h. A chair?<br>i. A bed?<br>j. A sofa?<br>k. A cupboard?<br>l. An air conditioner?<br>m. An electric iron?<br>n. A generator?<br>o. A fan?	j. Yes……1 No……..2<br>k. Yes……1 No……..2<br>l. Yes……1 No……..2<br>m. Yes……1 No……..2<br>n. Yes……1 No……..2<br>o. Yes……1 No……..2	

THANK THE RESPONDENT AND END THE INTERVIEW

QUESTIONNAIRE B2

**QUESTION NO./NAME**	**QUESTIONS AND FILTERS**	**CODING CATEGORIES**	**SKIP**
SCHOOL	Have you ever attended school?<br><br>ENUMERATOR: THIS QUESTIONS REFERS TO FORMAL SCHOOLING ONLY	No……0<br>Yes…….1	→ ETHN
LEVEL	What is the highest level of school you attended:<br>primary, secondary, or higher?	Primary…..1<br>Secondary…….2<br>Higher…….3	
GRADE	What is the highest (class/year) you completed at that level?<br><br>ENTER 00 IF DID NOT COMPLETE EVEN ONE YEAR AT THAT LEVEL	Class/Year |_|	
ETHN	What is your ethnic group?	1 Ekoi<br>2 Fulani<br>3 Hausa<br>4 Ibibio<br>5 Igala<br>6 Igbo<br>7 Ijaw/Izon<br>8 Kanuri/Beriberi<br>9 Tiv<br>10 Yoruba<br>96 Other<br>98 Don't know<br>999 Missing	
RELIG	What is your religion?	catholic . . . . 1<br>other christian . . . . 2<br>islam . . . . 3<br>traditionalist . . . . 4<br>other [specify: ……………….…] 6	
PREGCK	Are you pregnant now?	yes……1<br>no…….0<br>says she can't get pregnant …3<br><br>unsure…………8	→ IF 1 OR 3, THANK RESPONDENT AND END INTERVIEW
FERTINT_ONEQ	If you could fully control whether you got pregnant, and could do so without you or your partner doing anything specifically for you to avoid getting pregnant, would you personally want to <u>avoid</u> getting pregnant, at least in the next two years?	Yes…………………1<br>No…………………..0<br>SAYS SHE CAN'T GET PREGNANT 3	→ THANK RESPONDENT AND END INTERVIEW
FERTVALUE	How important would you say that it is for a woman to have children?<br><br>ENUMERATOR: READ ALL POSSIBLE ANSWERS ALLOWED AND RECORD ONLY ONE.	The most important thing a woman can do in her life…………………………1<br>Very important but not the only important thing a woman can do in her life…………2<br>Neither important nor unimportant……….3<br>Unimportant………………………………..4	

[NEW SCREEN]

Now I am going to ask questions, but I would like you to not tell me the answers directly. Instead, I am going to give you some vouchers that will allow you to answer the questions without me knowing what the answer is. Only the people analysing the data far away from here and who have never met you will know what your answer is.

MATERIALS NEEDED NOW:

1. ONE ENVELOPE MARKED WITH A “T” WHICH CONTAINS 4 WHITE COUPONS AND 4 BLACK COUPONS AND
2. THREE UNMARKED SEALED ENVELOPES WHICH EACH CONTAIN 9 WHITE COUPONS AND 9 BLACK COUPONS (ONLY 8 OF EACH ARE REQUIRED, THE 9TH COUPONS ARE NEEDED IN CASE THE RESPONDENTS ACCIDENTALLY TEARS A NUMBER OF THE CODE)

[NEW SCREEN]

COLOURBOX TRAINING:

ENUMERATOR GIVES THE RESPONDENT THE TRAINING VOUCHERS IN AN ENVELOPPE MARKED WITH A “T”.

Before we start, we will first do a training together with the training vouchers I have given you (T).

Just for this training, I will take the envelope with a T on it and I will place the coupons on the table, so that I can see if you understand the process.

I am going to ask you questions whose answer is always either YES or NO. YES corresponds to the colour white and NO to the colour black. Instead of answering me with YES or NO, I will ask you to take the coupon with the colour that corresponds to the answer, to tear it up and give me the code with the numbers.

(ENUMERATOR POINTS TO THE WHITE BOX OF THE VISUAL AID AND SAYS “YES” AND POINTS TO THE BLACK BOX THE VISUAL AID AND SAYS “NO”. ENUMERATOR REPEATS THE OPTIONS SLOWLY TWICE).

[NEXT SCREEN]

For instance, if I ask you “Are you female?”

Find the right coupon for your answer, tear the coupon along the dotted line, and give the part with the 6-digits code to me [ENUMERATOR DEMONSTRATES]. I will key in this code into the system like this [ENUMERATOR ENTERS 6-DIGIT CODE INTO TABLET]. And then hand it back to you like this. Please use a new coupon for the next question.

Please be very careful when tearing the coupon along the line. It may help to fold it first. The secret code must be legible so I can read it clearly. Please never give me a code with part or all the colour code still next to it as otherwise I would know what your answer is.

[NEXT SCREEN]

Now if I ask you: “Are you male?”

Please tear the right coupon and give the 6-digits code to me [ENUMERATOR DEMONSTRATES]. I will key in this code into the system like this [ENUMERATOR ENTERS 6-DIGIT CODE INTO TABLET]. And then hand it back to you like this.

[NEXT SCREEN]

Now, have a try at answering two practice questions yourself, but where I can see your answers so I can tell you if you are doing it right.

[NEXT SCREEN]

COLOURBOX_TR1	Are we in Nigeria?<br><br>ENUMERATOR: THE RESPONDENT SHOULD TEAR A WHITE COUPON. IF NOT, SAY GENTLY:<br><br>I expected you to tear a white coupon because we are in Nigeria and white means “yes”.	ENTER NUMBER ON VOUCHER: ☐☐☐☐☐☐<br><br>CAPI NOTES: PLEASE EMBED CHECKS TO WARN THE ENUMERATOR WHEN THE CODE IS INVALID.	

COLOURBOX_TR2	Are we in Lagos?<br><br>ENUMERATOR: THE RESPONDENT SHOULD TEAR A WHITE COUPON. IF NOT, SAY GENTLY:<br><br>I expected you to tear a black coupon because we are not in Lagos and black means "no".	ENTER NUMBER ON VOUCHER:<br>☐☐☐☐☐☐<br><br>CAPI NOTES: PLEASE EMBED CHECKS TO WARN THE ENUMERATOR WHEN THE CODE IS INVALID	

[NEXT SCREEN]

Please put the training coupons away. We will not use them further.

Now we will begin with the real session.

ENUMERATOR PRESENTS A CHOICE OF 3 SEALED, UNLABELLED ENVELOPES TO THE RESPONDENT.

Take a sealed envelope. Do not show me the contents.

You will use a new secret code for each new question. There are 8 questions in total, including two warm-up questions at the start so you can practice using the coupons without me seeing your answers. Remember that you will take away all the coupons and codes with you after the survey so there will be no way for me to work out which coupons you have used or not used.

I will turn away from you throughout this section to provide you with more privacy.

Are we ready to get started? We will now begin with the questions.

[NEXT SCREEN]

COLOURBOX_TR3	Are you more than 80 years old?	ENTER NUMBER ON VOUCHER:<br>☐☐☐☐☐☐<br><br>CAPI NOTES: PLEASE EMBED CHECKS TO WARN THE ENUMERATOR WHEN THE CODE IS NOT EXACTLY 6-DIGIT LONG.	
COLOURBOX_TR4	Are you less than 60 years old?	ENTER NUMBER ON VOUCHER:<br>☐☐☐☐☐☐<br><br>CAPI NOTES: PLEASE EMBED CHECKS TO WARN THE ENUMERATOR WHEN THE CODE IS NOT EXACTLY 6-DIGIT LONG.	
COLOURBOX1	Please remember to use a secret code to answer my questions rather than responding aloud.<br><br>If you could fully control whether you got pregnant, and could do so without you or your partner doing anything specifically for you to avoid getting pregnant, would you personally want to avoid getting pregnant, at least in the next two years?	ENTER NUMBER ON VOUCHER:<br>☐☐☐☐☐☐<br><br>RE-ENTER NUMBER ON VOUCHER:<br>☐☐☐☐☐☐	
COLOURBOX2	If you got pregnant soon, do you think you would you feel happy about it?	ENTER NUMBER ON VOUCHER:<br>☐☐☐☐☐☐<br><br>CAPI NOTES: PLEASE EMBED CHECKS TO WARN THE ENUMERATOR WHEN THE CODE IS NOT EXACTLY 6-DIGIT LONG.	
COLOURBOX3	If you got pregnant soon, do you think it would make your life better?	ENTER NUMBER ON VOUCHER:<br>☐☐☐☐☐☐<br><br>CAPI NOTES: PLEASE EMBED CHECKS TO WARN THE ENUMERATOR WHEN THE CODE IS NOT EXACTLY 6-DIGIT LONG.	
COLOURBOX4	Please remember to use a secret code to answer my questions rather than responding aloud.	ENTER NUMBER ON VOUCHER:<br>☐☐☐☐☐☐	

	If your husband/partner could fully control whether you got pregnant, and could do so without you or him doing anything specifically for you to avoid getting pregnant, do you think that he personally would prefer for you NOT to get pregnant, at least in the coming two years?	RE-ENTER NUMBER ON VOUCHER: ☐☐☐☐☐☐	
COLOURBOX5	If you got pregnant soon, do you think your husband would feel happy about it?	ENTER NUMBER ON VOUCHER: ☐☐☐☐☐☐<br><br>CAPI NOTES: PLEASE EMBED CHECKS TO WARN THE ENUMERATOR WHEN THE CODE IS NOT EXACTLY 6-DIGIT LONG.	
COLOURBOX6	If you got pregnant soon, do you think it would make your husband's life better?	ENTER NUMBER ON VOUCHER: ☐☐☐☐☐☐<br><br>CAPI NOTES: PLEASE EMBED CHECKS TO WARN THE ENUMERATOR WHEN THE CODE IS NOT EXACTLY 6-DIGIT LONG.	

Now I would like to ask a few questions about facilities in your household.

QUESTION NO./NAME	QUESTIONS AND FILTERS	CODING CATEGORIES	SKIP
WATER1	What is the main source of drinking water for members of your household?	**PIPED WATER**<br>PIPED INTO DWELLING . . . . 11<br>PIPED TO YARD/PLOT . . . . 12<br>PIPED TO NEIGHBOR . . . . 13<br><br>---<br><br>PUBLIC TAP/STANDPIPE . . . . 14<br><br>TUBE WELL OR BOREHOLE . . . . 21<br>**DUG WELL**<br>PROTECTED WELL . . . . 31<br>UNPROTECTED WELL . . . . 32<br>**WATER FROM SPRING**<br>PROTECTED SPRING . . . . 41 103<br>UNPROTECTED SPRING . . . . 42<br>RAINWATER . . . . 51<br>TANKER TRUCK . . . . 61<br>CART WITH SMALL TANK . . . . 71<br>SURFACE WATER (RIVER/DAM/ LAKE/POND/STREAM/CANAL/ IRRIGATION CHANNEL) . . . . 81<br><br>---<br><br>BOTTLED WATER . . . . 91<br>SACHET WATER . . . . 92<br><br>OTHER 96	→TOILET1<br>→TOILET1<br>→TOILET1<br><br>-----------------------------------<br><br>IF ANSWERS 14 TO 81 →WATER2<br><br><br><br><br><br><br><br><br>→ WATER2
WATER2	Where is that water source located?	IN OWN DWELLING . . . . 1<br>IN OWN YARD/PLOT . . . . 2<br>ELSEWHERE 3	→TOILET1<br>→TOILET1
WATER3	How long does it take to go there, get water, and come back?	|_|_|_| MINUTES<br>DON'T KNOW 998	
TOILET1	What kind of toilet facility do members of your household usually use?	**FLUSH OR POUR FLUSH TOILET**<br>FLUSH TO PIPED SEWER SYSTEM . . . . 11<br>FLUSH TO SEPTIC TANK . . . . 12<br>FLUSH TO PIT LATRINE . . . . 13<br>FLUSH TO SOMEWHERE ELSE . . . . 14<br>FLUSH, DON'T KNOW WHERE . . . . 15<br><br>**PIT LATRINE**<br>VENTILATED IMPROVED PIT LATRINE 21	

	IF NOT POSSIBLE TO DETERMINE, ASK PERMISSION TO OBSERVE THE FACILITY.	PIT LATRINE WITH SLAB . . . . 22<br>PIT LATRINE WITHOUT SLAB/OPEN PIT . . . . . 23<br>COMPOSTING TOILET . . . . 31<br>BUCKET TOILET . . . . 41<br>HANGING TOILET/HANGING LATRINE . . . . 51<br>NO FACILITY/BUSH/FIELD . . . . 61<br><br>OTHER [SPECIFY:…………………………….]……………………96	
TOILET2	Do you share this toilet facility with other households?	Yes……..1<br>No………0	
HHITEM	Does your household have:<br><br>p. Electricity?<br>q. A radio?<br>r. A television?<br>s. A non-mobile telephone?<br>t. A computer?<br>u. A refrigerator?<br>v. A table?<br>w. A chair?<br>x. A bed?<br>y. A sofa?<br>z. A cupboard?<br>aa. An air conditioner?<br>bb. An electric iron?<br>cc. A generator?<br>dd. A fan?	p. Yes……1 No……..2<br>q. Yes……1 No……..2<br>r. Yes……1 No……..2<br>s. Yes……1 No……..2<br>t. Yes……1 No……..2<br>u. Yes……1 No……..2<br>v. Yes……1 No……..2<br>w. Yes……1 No……..2<br>x. Yes……1 No……..2<br>y. Yes……1 No……..2<br>z. Yes……1 No……..2<br>aa. Yes……1 No……..2<br>bb. Yes……1 No……..2<br>cc. Yes……1 No……..2<br>dd. Yes……1 No……..2	

THANK THE RESPONDENT AND END THE INTERVIEW

FERTILITY AND HEALTH EXPECTATIONS SURVEY
UNIVERSITY OF BRISTOL

2023 VALIDATION QUESTIONNAIRE

**IDENTIFICATION**	
STATE:	
LOCAL GOVT. AREA:	
LOCALITY:	
ENUMERATION AREA:	
CLUSTER NUMBER:	
NAME OF HOUSEHOLD HEAD:	
HOUSEHOLD NUMBER (SAME AS IN LISTING EXERCISE):	
NAME AND LINE NUMBER OF WOMAN (FROM LISTING EXERCISE):	
TELEPHONE NUMBER 1:	TELEPHONE NUMBER 2:

INTERVIEWER'S VISITS				
	1	2	3	FINAL VISIT
DATE:				DAY ………..<br>MONTH …………<br>YEAR………..<br><br>INTERVIEW NO.<br>RESULT*
INTERVIEWER'S NAME:				
RESULT (ENUMERATOR: ARE YOU ABLE TO INDENTIFY AND INTERVIEW THE CORRECT RESPONDENT?)*				
NEXT VISIT: DATE<br>TIME				TOTAL NUMBER OF VISITS:

*RESULT CODES:
1. Yes, respondent can be found and is fit to answer
2. No, household has permanently left
3. No, household cannot be found after three visits
4. No, household still there but respondent has permanently left the household
5. No, household still there but respondent cannot be found after three visits
6. No, respondent is permanently incapacitated
7. No, respondent is deceased
8. No, household or individual refuses to answer

ENUMERATOR: PROCEED WITH THE INTERVIEW EVEN IF YOU FIND OUT THAT THE RESPONDENT IS INELIGIBLE FOR A FULL INTERVIEW (DUE TO BEING PREGNANT, ETC…)

LANGUAGE OF QUESTIONNAIRE**:	LANGUAGE OF INTERVIEW**:	NATIVE LANGUAGE OF RESPONDENT**:	TRANSLATOR USED:
|_|_|	|_|_|	|_|_|	YES/NO
**LANGUAGE CODES: 01 ENGLISH 03 YORUBA 02 HAUSA			

	SUPERVISOR	FIELD EDITOR
NAME		
NUMBER		

INTRODUCTION AND CONSENT

Hello. My name is ____. I am working with National Population Commission. You may recall that one of my colleagues visited you nearly a year ago. We are conducting a survey about fertility and health in five states of Nigeria. The information we collect will help researchers understand what people in these areas think about ways of planning the size of their family. The results from this study will then be shared with the government of Nigeria and other nearby countries to inform their plans regarding family health services. You were selected for a short survey which usually takes up to 20 minutes. All of the answers you give will be confidential and will not be shared with anyone other than members of our survey team. You don't have to be in the survey, but we hope you will agree to answer the questions since your views are important. If I ask you any question you don't want to answer, just let me know and I will go on to the next question or you can stop the interview at any time.

In case you need more information about the survey, you may contact the person listed on this card.

Do you have any questions?
May I begin the interview now?

<table>
<tr><td>RESPONDENT AGREES TO BE INTERVIEWED |_|</td><td>SIGNATURE OF RESPONDENT:</td><td rowspan="2"></td></tr>
<tr><td>RESPONDENT DOES NOT AGREE TO BE INTERVIEWED |_|</td><td></td></tr>
<tr><td>DATE: |__|__|__|</td><td>SIGNATURE OF INTERVIEWER:</td><td rowspan="2"></td></tr>
<tr><td></td><td></td></tr>
</table>

INTERVIEWER: THE TEXT IN CAPITAL LETTERS IS INTENDED FOR YOU ONLY. DO NOT READ IT ALOUD.

QUESTIONNAIRE

QUESTION NO./NAME	QUESTIONS AND FILTERS	CODING CATEGORIES	SKIP
CONSENT	ENUMERATOR: READ INTRODUCTION AND CONSENT?	YES….1<br>NO…...0<br>(CAPI SHOULD NOT PROCEED IF DOES NOT SELECT YES)	
BEGN_INTV	May I begin the interview?	YES….1<br>NO…...0	→HUSB_WIFE<br>→THANK RESPONDENT AND END INTERVIEW
HUSB_WIFE	Are you currently married or living with a man as husband and wife?	YES….1<br>NO…...0	
AGECHCK2	How old were you at your last birthday?	|_|_|	IF ANSWERS A NUMBER OF YEARS ENDING WITH 0 OR 5, ASK AGE_HEAPING. IF NOT, SKIP TO PREGCK
AGE_HEAPING	Are you exactly [AGECHCK2 RESPONSE], or are you a bit older or a bit younger? How old were you at your last birthday exactly?<br><br>ENUMERATOR: IF THE RESPONDENT IS UNSURE, HELP THEM WORK OUT THEIR AGE BY ASKING FOR THEIR BIRTH YEAR/MONTH.	|_|_|	
PREGCK	Are you pregnant now?	yes……1<br>no…….0<br>unsure………….8<br>refuses to answer…..9<br>says she can't get pregnant because of something **irreversible** (never fertile, menopause, hysterectomy, female sterilization)….333	→ IF 1 OR 333, THANK RESPONDENT AND END INTERVIEW

QUESTION NO./NAME	QUESTIONS AND FILTERS	CODING CATEGORIES	SKIP
WANTS2	Now I have some questions about the future. If you could fully control whether you got pregnant, and could do so without you or your partner doing anything specifically for you to avoid getting pregnant, would you personally like to have (a/another) child, or would you prefer not to have any (more) children?	have another child . . . . 1<br>no more . . . . 2<br>says she can't get pregnant……3<br>undecided/don't know 8	
	CAPI CODING NOTES:<br>- FOR KNOWS_1 AND KNOWS_2, RESPONDENTS MUST BE RANDOMIZED INTO GROUP 1 AND GROUP 2 WITH PROBABILITY 0.5, WITHIN 10 GROUPS DEFINED BY: AGE GROUP (18-24, 25-29, 30-34, 35-39, 40-44), POLYGAMOUS/MONOGAMOUS.<br>- DO NOT USE GROUPS X AND Y FROM THE LIST EXPERIMENT. THE RANDOMIZATION METHOD IS THE SAME, BUT YOU SHOULD REPEAT THE PROCESS (WHICH WILL LEAD TO DIFFERENT SPLITS OF THE SAMPLE).<br>- EACH RESPONDENT IS EITHER ASKED THE SET OF KNOWS_1 QUESTIONS **OR** THE SET OF KNOWS_2 QUESTIONS, NEVER BOTH.		
	IF GROUP = 1, ASK KNOWS_1.<br><br>IF GROUP = 2, ASK KNOWS_2.		

KNOWS_1	Now I would like to talk about family planning - the various ways or methods that a couple can use to delay or avoid a pregnancy.<br><br>Have you ever heard of (METHOD)?<br><br>ENUMERATOR: IF THE RESPONDENT SAYS NO AT FIRST, READ THE PROBE SLOWLY TO MAKE SURE SHE UNDERSTANDS, SHOW HER THE PICTURE IN YOUR BOOKLET AND TRY TO EXPLAIN WITH OTHER WORDS AND TERMS WHICH PEOPLE MIGHT USE TO REFER TO THE METHOD.		
A	Female Sterilization.<br>PROBE: Women can have an operation to avoid having any more children.	yes……1<br>no…….0	
B	Male Sterilization.<br>PROBE: Men can have an operation to avoid having any more children.	yes……1<br>no…….0	
C	IUD.<br>PROBE: Women can have a loop or coil placed inside them by a doctor or a nurse which can prevent pregnancy for one or more years.	yes……1<br>no…….0	
D	Injectables.<br>PROBE: Women can have an injection by a health provider that stops<br>them from becoming pregnant for one or more months.	yes……1<br>no…….0	
E	Implants.<br>PROBE: Women can have one or more small rods placed in their upper arm by a doctor or nurse which can prevent pregnancy for one<br>or more years.	yes……1<br>no…….0	
F	Pill.<br>PROBE: Women can take a pill every day to avoid becoming pregnant.	yes……1<br>no…….0	
G	Condom.<br>PROBE: Men can put a rubber sheath on their penis before sexual<br>intercourse.	yes……1<br>no…….0	
H	Female Condom.<br>PROBE: Women can place a sheath in their vagina before sexual<br>intercourse.	yes……1<br>no…….0	
I	Emergency Contraception.<br>PROBE: As an emergency measure, within three days after they have<br>unprotected sexual intercourse, women can take special pills to prevent pregnancy.	yes……1<br>no…….0	
K	Lactational Amenorrhea Method (LAM).<br>PROBE: Women can postpone the return of menstruation after a birth (and therefore remain unlikely to become pregnant) by breastfeeding frequently in the	yes……1<br>no…….0	

	first six months after a baby is born.		
L	Rhythm Method/ Standard Days Method.<br>PROBE: To avoid pregnancy, women do not have sexual intercourse on the days of the month they think they can get pregnant, or at least they use a condom on those days. Sometimes the woman uses a string of colored beads to know the days she can get pregnant.	yes……1<br>no…….0	
M	Withdrawal.<br>PROBE: Men can be careful and pull out before climax.	yes……1<br>no…….0	
KNOWS_2	Now I would like to talk about family planning - the various ways or methods that a couple can use to delay or avoid a pregnancy.<br><br>Have you ever heard of (METHOD)?<br><br>ENUMERATOR: SIMPLY ASK IF THE RESPONDENT HAS EVER HEARD OF THE METHOD. **DO NOT** PROBE IF SHE ANSWERS "NO".		
A	Female Sterilization.	yes……1<br>no…….0	
B	Male Sterilization.	yes……1<br>no…….0	
C	IUD.	yes……1<br>no…….0	
D	Injectables.	yes……1<br>no…….0	
E	Implants.	yes……1<br>no…….0	
F	Pill.	yes……1<br>no…….0	
G	Condom.	yes……1<br>no…….0	
H	Female Condom.	yes……1<br>no…….0	
I	Emergency Contraception.	yes……1<br>no…….0	
K	Lactational Amenorrhea Method (LAM).	yes……1<br>no…….0	
L	Rhythm Method/ Standard Days Method.	yes……1<br>no…….0	
M	Withdrawal.	yes……1<br>no…….0	

KNOWLEDGE_PREG	For each of the following methods, how well informed do you think you are about how effective it is in avoiding pregnancy?<br><br>ENUMERATOR READS ALL POSSIBLE ANSWERS ALOUD AND SELECTS ONLY ONE.<br><br>IF THE RESPONDENT ANSWERS THAT SHE HAS NEVER HEARD OF THE METHOD, ASK HER AGAIN POLITELY TO CHOOSE ONE OF THE POSSIBLE RESPONSES. A RESPONDENT MAY NOT HAVE HEARD OF A PARTICULAR METHOD BUT FEEL THAT SHE KNOWS OF ANOTHER METHOD THAT SHE THINKS IS SIMILAR.	CAPI NOTES: ASK ALL QUESTIONS IRRESPECTIVE OF ANSWERS TO KNOWS_1 OR KNOWS_2.	
C	IUD	Very well informed….3<br><br>Has some idea but not very well informed……3<br>Has no idea at all…..4	
D	Implants	Very well informed….3<br><br>Has some idea but not very well informed……3<br>Has no idea at all…..4	
E	Injections	Very well informed….3<br><br>Has some idea but not very well informed……3<br>Has no idea at all…..4	
F	Pill	Very well informed….3<br><br>Has some idea but not very well informed……3<br>Has no idea at all…..4	
G	Condoms	Very well informed….3<br><br>Has some idea but not very well informed……3<br>Has no idea at all…..4	
K	Lactational Amenorrhea (LAM)	Very well informed….3<br><br>Has some idea but not very well informed……3<br>Has no idea at all…..4	
L	Rhythm Method/ Standard Days Method.	Very well informed….3<br><br>Has some idea but not very well informed……3<br>Has no idea at all…..4	
M	Withdrawal	Very well informed….3<br><br>Has some idea but not very well informed……3<br>Has no idea at all…..4	
KNOWLEDGE_HEALTH	For each of the following methods, how well informed do you think you are about whether there are any health risks to using each of these methods?<br><br>IF THE RESPONDENT ANSWERS THAT SHE		

	HAS NEVER HEARD OF THE METHOD OR THAT THERE IS NO HEALTH RISK, ASK HER AGAIN POLITELY TO CHOOSE ONE OF THE POSSIBLE RESPONSES. A RESPONDENT MAY NOT HAVE HEARD OF A PARTICULAR METHOD BUT FEEL THAT SHE KNOWS OF ANOTHER METHOD THAT SHE THINKS IS SIMILAR.		
C	IUD	Very well informed….3<br><br>Has some idea but not very well informed……3<br>Has no idea at all…..4	
D	Implants	Very well informed….3<br><br>Has some idea but not very well informed……3<br>Has no idea at all…..4	
E	Injections	Very well informed….3<br><br>Has some idea but not very well informed……3<br>Has no idea at all…..4	
F	Pill	Very well informed….3<br><br>Has some idea but not very well informed……3<br>Has no idea at all…..4	
G	Condoms	Very well informed….3<br><br>Has some idea but not very well informed……3<br>Has no idea at all…..4	
K	Lactational Amenorrhea (LAM)	Very well informed….3<br><br>Has some idea but not very well informed……3<br>Has no idea at all…..4	
L	Rhythm Method/ Standard Days Method.	Very well informed….3<br><br>Has some idea but not very well informed……3<br>Has no idea at all…..4	
M	Withdrawal	Very well informed….3<br><br>Has some idea but not very well informed……3<br>Has no idea at all…..4	
CONSIDERATION	I am going to read out the name of each of the methods you have heard of. Is there any method which you would never consider using even if you and your husband really wanted to delay or avoid pregnancy and no other method was available to you?<br><br>[ASK ABOUT ALL METHODS FOR WHICH THE RESPONDENT ANSWERED “YES” TO THE CORRESPONDING KNOWS_1 OR KNOWS_2 QUESTION]		

C	KNOWS_1_C=1? IF YES, ASK:<br><br>IUD	**Could consider** using this method…….0<br>**Would never** consider using this method..1<br>Would never consider using **any** method...8<br>Refuses to answer…….9<br><br>ENUMERATOR READS ALL POSSIBLE ANSWERS ALOUD EXCEPT "REFUSES TO ANSWER"	
D	KNOWS_1_D=1? IF YES, ASK:<br><br>Implants	**Could consider** using this method…….0<br>**Would never** consider using this method..1<br>Would never consider using **any** method...8<br>Refuses to answer…….9<br><br>ENUMERATOR READS ALL POSSIBLE ANSWERS ALOUD EXCEPT "REFUSES TO ANSWER"	
E	KNOWS_1_E=1? IF YES, ASK:<br><br>Injections	**Could consider** using this method…….0<br>**Would never** consider using this method..1<br>Would never consider using **any** method...8<br>Refuses to answer…….9<br><br>ENUMERATOR READS ALL POSSIBLE ANSWERS ALOUD EXCEPT "REFUSES TO ANSWER"	
F	KNOWS_1_F=1? IF YES, ASK:<br><br>Pill	**Could consider** using this method…….0<br>**Would never** consider using this method..1<br>Would never consider using **any** method...8<br>Refuses to answer…….9<br><br>ENUMERATOR READS ALL POSSIBLE ANSWERS ALOUD EXCEPT "REFUSES TO ANSWER"	
G	KNOWS_1_G=1? IF YES, ASK:<br><br>Condoms	**Could consider** using this method…….0<br>**Would never** consider using this method..1<br>Would never consider using **any** method...8<br>Refuses to answer…….9<br><br>ENUMERATOR READS ALL POSSIBLE ANSWERS ALOUD EXCEPT "REFUSES TO ANSWER"	
K	KNOWS_1_K=1? IF YES, ASK:<br><br>Lactational Amenorrhea (LAM)	**Could consider** using this method…….0<br>**Would never** consider using this method..1<br>Would never consider using **any** method...8<br>Refuses to answer…….9<br><br>ENUMERATOR READS ALL POSSIBLE ANSWERS ALOUD EXCEPT "REFUSES TO ANSWER"	
L	KNOWS_1_L=1? IF YES, ASK:<br><br>Rhythm Method/ Standard Days Method.	**Could consider** using this method…….0<br>**Would never** consider using this method..1<br>Would never consider using **any** method...8<br>Refuses to answer…….9<br><br>ENUMERATOR READS ALL POSSIBLE ANSWERS ALOUD EXCEPT "REFUSES TO ANSWER"	
M	KNOWS_1_M=1? IF YES, ASK:<br><br>Withdrawal	**Could consider** using this method…….0<br>**Would never** consider using this method..1<br>Would never consider using **any** method...8<br>Refuses to answer…….9<br><br>ENUMERATOR READS ALL POSSIBLE ANSWERS ALOUD EXCEPT "REFUSES TO ANSWER"	
BIN_12MONTHS	Have you used anything or tried in any way to delay or avoid getting pregnant at any point in the past 12 months?	yes……1<br>no…….0	
BINCURRENT	Are you or your partner currently doing something or using any method to delay or avoid getting pregnant?	yes……1<br>no…….0	→CONDOM

TRAD_NEW_DHS	Just to check, are you or your partner doing any of the following to avoid pregnancy: deliberately avoiding sex on certain days, using a condom, using withdrawal or using emergency contraception?	yes……1<br>no…….0	
CONDOM	Sometimes, people use condoms for other reasons than to delay or avoid getting pregnant. In the past 12 months, have you used condoms mainly for another reason than to delay or avoid getting pregnant?	yes……1<br>no…….0<br>Doesn't know what condoms are…..8<br>Refuses to answer…………9	
BINEVER	Have you ever used anything or tried in any way to delay or avoid getting pregnant?	yes……1<br>no…….0	

**QUESTION NO./NAME**	**QUESTIONS AND FILTERS**	**CODING CATEGORIES**	**SKIP**
	CAPI CODING NOTES:<br>- FOR 401_V1 AND 401_V 2, RESPONDENTS MUST BE RANDOMIZED INTO GROUP V1 AND GROUP V2 WITH PROBABILITY 0.5, WITHIN 10 GROUPS DEFINED BY: AGE GROUP (18-24, 25-29, 30-34, 35-39, 40-44), POLYGAMOUS/MONOGAMOUS.<br>- DO NOT USE GROUPS X AND Y FROM THE LIST EXPERIMENT OR GROUPS 1 AND 2 USED TO RANDOMIZE KNOWS_1 AND KNOWS_2. THE RANDOMIZATION METHOD IS THE SAME, BUT YOU SHOULD REPEAT THE PROCESS (WHICH WILL LEAD TO DIFFERENT SPLITS OF THE SAMPLE). EACH RESPONDENT IS EITHER ASKED THE SET OF 401_V1 QUESTIONS **OR** THE SET OF 401_V2 QUESTIONS, NEVER BOTH.		
	IF GROUP = V1, ASK 401_V1.<br><br>IF GROUP = V2, ASK 401_V2.		
401_V1	**If it was only up to you and no one else had a say or an opinion about what you did**, please can you tell me which of the following options would be your preferred one right now?<br><br>CAPI PROGRAMMING NOTES: THE OPTIONS BELOW SHOULD ONLY BE LISTED IF THE RESPONDENT SAYS SHE KNOWS THEM IN THE CORRESPONDING `KNOWS-METHOD CODE' QUESTION.<br><br>READ THE FOLLOWING OPTIONS AND SHOW THE FLASHCARDS TO HELP THE RESPONDENT ORDER PREFERENCES (DO NOT READ THE CODES). **MAKE SURE TO INCLUDE THE "NO METHOD" FLASHCARD.**<br><br>Response codes:<br>1 "female sterilization"<br>2 "male sterilization"<br>3 "IUD"<br>4 "Injectables"<br>5 "Implants"<br>6 "Pill"		

	7 "Condom"<br>8 "Female condom"<br>9 "Emergency contraception"<br>10 "Lactational amenorrhea method"<br>11 "Rhythm/Standard days method"<br>12 "Withdrawal"<br>13 "Other method"<br>14 - Using no contraceptive method at all<br>99 – no preference at all<br>999 – doesn't know any option not yet chosen as first- or second- preference<br><br>THEN RECORD THE FIRST THREE PREFERRED OPTIONS IN THE ORDER OF PREFERENCE GIVEN BY THE RESPONDENT.		
1	Most preferred option:	|__|	
2	Second preferred option:	|__|	
3	Third preferred option:	|__|	
401_V2	**If it was only up to you and no one else had a say or an opinion about what you did**, please can you tell me which of the following contraceptive methods would be your preferred one right now?<br><br>CAPI PROGRAMMING NOTES: THE OPTIONS BELOW SHOULD ONLY BE LISTED IF THE RESPONDENT SAYS SHE KNOWS THEM IN THE CORRESPONDING `KNOWS-METHOD CODE' QUESTION. THE METHOD CODE IS SHOWN BELOW BEFORE THE METHOD NAME. PLEASE MAKE SURE THE SAME OPTION CANNOT BE ENTERED IN MORE THAN ONE PREFERENCE ORDER.<br><br>READ THE FOLLOWING OPTIONS AND SHOW THE FLASHCARDS TO HELP THE RESPONDENT ORDER PREFERENCES (DO NOT READ THE CODES). **DO NOT INCLUDE THE "NO METHOD" FLASHCARD.**<br><br>Response codes:<br>1 "female sterilization"<br>2 "male sterilization"<br>3 "IUD"<br>4 "Injectables"<br>5 "Implants"<br>6 "Pill"<br>7 "Condom"<br>8 "Female condom"<br>9 "Emergency contraception"<br>10 "Lactational amenorrhea method"<br>11 "Rhythm/Standard days method"<br>12 "Withdrawal"<br>13 "Other method"<br><br>99 – no preference at all<br>999 – doesn't know any method (or doesn't know any method not yet chosen as first- or second- preference)<br><br>THEN RECORD THE FIRST THREE PREFERRED OPTIONS IN THE ORDER OF PREFERENCE GIVEN BY THE RESPONDENT.		
1	Most preferred option:	|__|	
2	Second preferred option:	|__|	
3	Third preferred option:	|__|	

Final Section (Adapted from 2022 Questionnaire B1)

<table>
<tr><td>INTRO</td><td colspan="2">I am going to read a list of statements aloud to you. Please tell me how many of these statements are true without telling me which ones are true and which ones are not (, so nobody can ever know which statement applies to you and which does not)</td><td></td></tr>
<tr><td>LIST EXP. 1</td><td colspan="2">To help you count the number of statements you agree with, please first place one hand behind your back and make a fist. If you agree with the statement I am reading, please raise one finger like this (ENUMERATOR DEMONSTRATES), keeping your hand behind your back. If you do not agree with this statement, do nothing.

Once all the statements have been read, you should have as many fingers raised behind your back as statements you agree with. At the end of reading all the statements, I will ask you to show me your hand so I know how many statements you agree with.

Here is an example. Consider the following statements:

You like papaya.
You like watermelon.
You like pineapple.

Imagine that you only like pineapple. If you heard these three statements, then you would make a fist behind your back and raise only one finger when you hear the last statement. If you liked two out of these three types of fruit, you would instead have two fingers raised after I finish reading the statements.</td><td></td></tr>
<tr><td>CONCLUSION</td><td colspan="2">I will now read the statements for the study. Please (do not tell me whether you agree or disagree with one particular statement or show any sign that you agree or not,) just show me your hand at the end so I know how many statements you agree with.</td><td></td></tr>
<tr><td>ASK Group X:</td><td>1. You hope that your children (if/when you have them) stay healthy.
2. In the past 12 months, you have read a message about family planning on a poster or heard a message about family planning on the radio.
3. You think that family planning decisions should be made jointly by husband and wife.
4. You think that 40 is a very good age for a woman to get married for the first time.</td><td>NUMBER OF TRUE STATEMENTS: |__|
(VALID ANSWERS: 0, 1, 2, 3, or 4)</td><td></td></tr>
<tr><td>ASK Group Y:</td><td>1. You hope that your children (if/when you have them) stay healthy.
2. In the past 12 months, you have read a message about family planning on a poster or heard a message about family planning on the radio.
3. You think that family planning decisions should be made jointly by husband and wife.
4. You think that 40 is a very good age for a woman to get married for the first time.
5. If you could fully control whether you got pregnant, and could do so without you or your partner doing anything specifically for</td><td>NUMBER OF TRUE STATEMENTS: |__|
(VALID ANSWERS: 0, 1, 2, 3, 4 or 5)</td><td></td></tr>
</table>

	you to avoid getting pregnant, personally you would want to **<u>avoid</u>** getting pregnant, at least in the next two years.		
FERTINT_ONEQ	Thank you for telling me how many statements you agree with. Now may I ask:<br><br>If you could fully control whether you got pregnant, and could do so without you or your partner doing anything specifically for you to avoid getting pregnant, would you personally want to **<u>avoid</u>** getting pregnant, at least in the next two years?	Yes…………………1<br>No…………………..0<br>Refuses to answer….9	
FERTSCALE	On a scale from 1 to 10, where 10 means that all you want in life right now is to get pregnant and 1 means that getting pregnant now would be the worst possible thing to happen to you, what number would best reflect how **you** would feel about getting pregnant right now?	RESPONSE: |_|_| (VALID RESPONSES ARE 1, 2, 3, 4, 5, 6, 7, 8, 9, OR 10)	
H_FERTSCALE	On a scale from 1 to 10, where 10 means that all your husband wants in life right now is for you to get pregnant and 1 means that you getting pregnant now would be the worst possible thing to happen to your husband, what number do you think would best reflect how your husband would feel about you getting pregnant right now?	RESPONSE: |_|_| (VALID RESPONSES ARE 1, 2, 3, 4, 5, 6, 7, 8, 9, OR 10)	

THANK THE RESPONDENT AND END THE INTERVIEW.
